# A Multifunctional Bread Rich in Beta Glucans and Low in Starch Improves Metabolic Control in Type 2 Diabetes: A Controlled Trial

**DOI:** 10.3390/nu9030297

**Published:** 2017-03-17

**Authors:** Paolo Tessari, Anna Lante

**Affiliations:** 1 Department of Medicine (DIMED), University of Padova, 35128 Padova PD, Italy; 2 Department of Agronomy, Food, Natural Resources, Animals & Environment (DAFNAE), University of Padova, 35123 Padova PD, Italy; anna.lante@unipd.it

**Keywords:** plasma glucose, beta glucan, starch, metabolic control, fibers

## Abstract

Design: Functional foods may be useful for people with diabetes. The soluble fibers beta glucans can modify starch digestion and improve postprandial glucose response. We analyzed the metabolic effects of a specifically designed ‘functional’ bread, low in starch, rich in fibers (7 g/100 g), with a beta glucan/starch ratio of (7.6:100, g/g), in people with type 2 diabetes mellitus. Methods*:* Clinical and metabolic data from two groups of age-, sex- and glycated hemoglobin-matched diabetic subjects, taking either the functional bread or regular white bread, over a roughly six-month observation period, were retrieved. Results: Bread intake did not change during the trial. The functional bread reduced glycated hemoglobin by ~0.5% (absolute units) vs. pre-treatment values (*p* = 0.028), and by ~0.6% vs. the control group (*p* = 0.027). Post-prandial and mean plasma glucose was decreased in the treatment group too. Body weight, blood pressure and plasma lipids did not change. The acceptance of the functional bread was good in the majority of subjects, except for taste. Conclusions: A starch-restricted, fiber-rich functional bread, with an increased beta glucan/starch ratio, improved long term metabolic control, and may be indicated in the dietary treatment of type 2 diabetes.

## 1. Introduction

### Background and Aims

Type 2 diabetes mellitus (T2DM) is a disease with high prevalence and increasing incidence. The world population currently affected by T2DM is about 400 million, and it is expected to grow to more than 600 million by 2040 [1]. The increase of T2DM is largely due to changes of lifestyle [2], i.e., excess dietary intake combined to low physical activity. Therefore, diet represents a cornerstone for both prevention and therapy of T2DM, and a tool to maintain plasma glucose values close to normal. 

Carbohydrates (CHO) are the key nutritional factors conditioning circulating glucose levels, particularly in the post-prandial state. In order to prevent excessive post-prandial glycemicexcursions, dietary CHO should be predominantly represented by starch (>90% of total CHO), with less than 10% by either mono- or di-saccharides [3]. Furthermore, since T2DM is associated with a constellation of conditions (obesity, dyslipidemia, hypertension) and risk factors (e.g., for cardiovascular disease) recognized in the concept of the ‘metabolic syndrome’ [4,5,6], food capable of addressing most, if not all these abnormalities, can be recommended. Thus, current understanding is that foods and diets for T2DM patients are considered, for the present, to be energy controlled, with a low glycemic index (GI), and with favorable effects on blood pressure and dyslipidemia. A reduction of cardiovascular (CV) risk and the prevention of long term diabetic complications are also expected to result from long-term use of such ‘ideal’ foods. 

Post-prandial glucose increments in diabetes may be blunted by foods containing starch and/or simple CHO with a slow absorption rate, thus resulting in a low GI. Besides by the CHO type itself, CHO absorption may be modulated by a variety of additional factors, such as starch characteristics (i.e., resistance to digestion), fiber type and content, and by the presence of other compounds that can decrease carbohydrate intestinal digestion and absorption. Conversely, the guidelines for dietary treatment of hypertension, obesity and dyslipidemia include a reduction of sodium and energy intakes, and a control of intestinal lipid absorption. Thus, targeting some, if not all, these risk factors, by means of functional foods seems to be both recommended and promising in the dietary management of both diabetes and the metabolic syndrome, through health-beneficial properties conveyed by natural components and/or by added ingredients [7].

Functional foods rich in natural fibers, such as beta glucans, are among those most-widely studied and employed. Beta glucans are naturally-occurring soluble fibers of some cereals (oats and barley), composed of mixed-linkage (1,3)(1,4)-β-d-glucose units, mainly consisting of the linear polysaccharide (1→3), (1→4)-β-d-glucan. Beta glucans increase viscosity in the upper digestive tract, thus reducing postprandial glucose and insulin responses [8]. An optimal beta glucan/starch ratio has been proposed as a method to decrease starch digestion in functional breads [9]. 

Bread is a staple food and a fundamental source of CHO worldwide in everyday diet. Therefore, the design of a bread with targeted functional properties could retain a major impact on nutrition and on metabolic parameters.

In this study, we examined the metabolic effects of long-term (roughly six months) substitution of regular white bread with a functional bread, starch-restricted and rich in fiber (mostly beta glucans), in the everyday diet of persons with type 2 diabetes. 

## 2. Methods

### 2.1. Study Design and Participants

In the context of a comprehensive analysis of clinical and biochemical parameters of people with type 2 diabetes mellitus, data from T2DM subjects of both sexes, collected at their regularly-scheduled outpatient visits at the Diabetes Center of the Padova University Hospital, Italy, were retrieved. This was an observational, controlled study with parallel groups, performed following recently published guidelines and format (http://www.consort-statement.org/). The subjects’ inclusion criteria were: a diabetes duration longer than 2 years; an unsatisfactory diabetes control (HbA_1_c values greater than 7%); age between 50 and 80 years; treatment with diet only, or diet plus oral hypoglycemic agents (OHA) and/or basal insulin; and a reported daily intake of at least 50 g CHO as either white bread, breadsticks or other bread substitutes. The patients were visited at 3–6 months intervals. 

### 2.2. Interventions

The patients had routinely been recommended to consume foods considered to be beneficial to peoples with diabetes, such as low calorie items, with CHO predominantly represented by starch, with a high fiber content and/or a low glycemic index, and low amounts of fats of animal origin. Furthermore, the patients had been informed about the availability in supermarkets, of flour products enriched with fibers and/or other compounds that could be beneficial to control glucose levels. They were encouraged to prepare homemade bread using any of these flour products, as a substitute of their usually consumed white bread made of refined flour. 

At subsequent examinations, the type of bread used by every subjects was recorded. Therefore, two groups of subjects could be selected over time: those who had shifted to the use of one of the functional bread types (‘functional bread’ group), and those who had been still using their regular white bread (‘control’ group). Among the former, the starting time of consumption of either the functional flour and/or bread, and its type, were retrieved and recorded. For the sake of homogeneity, we report here data of those subjects consuming only one type of these fiber-enriched products, i.e., a flour labelled Salus^®^ (produced by Ruggeri srl, Padova, Italy), that was the most largely used. These subjects were included into the ‘functional bread’ group. The data of the case-control group, i.e., that of patients who were consuming their regular bread, were collected in a similar fashion. The matching criteria with the ‘functional bread’ group were age, sex, absolute HbA_1_c values as well as absence of HbA_1_c changes between the two selected time intervals (i.e., approximately ~6–7 months. apart), greater than ±1% (absolute value). For both groups, other inclusion criteria were: no variation in both hypoglycemic and other drug therapy, and no major intercurrent diseases over the chosen observation periods. A total of eleven subjects in each group were selected.

The target observations interval after the ‘start’ time point was set between 3 and 12 months. Each patient had been advised to maintain his/her usual dietary habits and nutrient intake, except for the type of bread used. The 24-h recall method was used to assess dietary intake of the day before each visit. The data of each patients were handled rigorously in anonymous fashion. Of the 20 subjects originally enrolled into the ‘functional bread group’, only eleven completed the intended observational time period. The causes for drop-out were: insufficient bread intake (less than 40 g of starch equivalents per day, possibly preventing the detection of a significant effect by substitution with the functional bread); intercurrent diseases and/or changes of therapy; major dietary changes and/or failure to observe the standard recommendations for people with T2DM. 

As controls, two sets of data were used. First, each patient’s own HbA_1_c value(s) in the 3–12 month period antecedent either the start of consumption of the functional bread/flour, or a comparable period of use of the regular bread in the ‘control’ group, were recorded. These data are referred to as the ‘previous’ time points. Each patient was thus the control of him/herself. Second, data collected after use of either the ‘functional bread’ and of the ‘control bread’, as described above, recorded over the subsequent 3–12 months from the ‘start’ time point, were retrieved.

The subjects were either in the fasting state or had consumed their breakfast at home before coming to the Diabetes Centre. Breakfast composition was pretty reproducible in the same subject on the two/three visits, but different from patient to patient. In general, the patients consumed a medium-to-low-calorie Italian breakfast, consisting of milk, coffee (or tea), cereals (or bread slices), dressed with a thin layer of jam with a low sugar content, and/or one fruit, and/or one portion of yoghurt.

On the occasion of each visit, the patients’ glucose value (either in the fasting state or 2-h post-breakfast, the latter labeled as ‘post-prandial’ value), were determined by a reflectometer. These directly determined values were averaged with those retrieved from the home glucose monitoring of each patients, in the two–three days preceding the visit. Thus, both the fasting and post-breakfast data were the mean of these values.

The local Ethics Committee of Padova University Hospital had approved, as part of broader clinical data investigations in diabetes, the retrieval in anonymous form, of clinical and biochemical data from medical records of the diabetic patients (Approval N. 14809, released on 11 March 2016).

### 2.3. Bread Preparation

The functional bread was prepared at home by every subject using the instructions of the producer(s). Typically, a Moulinex bread machine (model. OW 3101, Moulinex, Groupe Seb Italia Spa, Milan, Italy) was employed. The composition of the functional bread (Salus^®^) flour and that of the refined flour used for the preparation of the control white bread, are reported on Table 1. The botanical source of the Beta-glucans was oat, as reported by the producers on the label of flour Salus (see Reg. (CE) n. 1924/2006 and Reg. (CE) n. 1160/2011). The mix of ingredients to prepare the functional bread was contained in packs of 500 g containing about 20 g of β-glucan and 200 g of starch. 

Bread baking was carried out using standard conditions for a white bread, as indicated by the manufacturer, as follows. Five hundred grams of the ingredient’s mixture and 430 mL of water were added into the bread machine’s pan. The bread maker took three hours to bake a loaf of bread. From about 450 g dough, 320–360 g cooked bread are obtained with a moisture percent ranging between 42%–43%. A bread loaf of 100 g contained about 2.3 g of β-glucans. 

In order to determine consumer acceptability, a simple 5-point hedonic scale (questionnaire) for each sensorial attribute, was used, where 5 was the highest (i.e., extremely positive) score (‘like very much’) and 1 the lowest (i.e., extremely negative) (‘dislike very much’). Participants were asked to rate odor, taste (salty and sweet), texture (soft and crisp) and general acceptance on the five-point Likert scale [10]. 

### 2.4. Outcome

The data of plasma glucose, HbA_1_c, lipids, blood pressure, and weight were retrieved from each subject’s medical records on the regularly scheduled visits. The methods of analyses were those standardized in the central analysis laboratory of the Padova University Hospital [11]. The post-prandial glucose values refer to measurements taken ~2 h after the meal (breakfast). The mean glucose concentrations resulting from the fasting and the post-prandial values was also calculated.

### 2.5. Sample Size and Statistical Analysis

By considering the primary end-point, i.e., a relative change of HbA_1_c of 0.6% (as absolute units) between the treated vs. the control group, and assuming a population mean of HbA_1_c of 8%, and an SD of 10% of the mean, a significant effect (α = 0.05, 1 − β = 0.80), would be attained by the sample size here studied. 

All data were expressed as means ± standard error (SE). The two-way analysis of variance (ANOVA) for repeated measurements, followed by post-hoc test, was employed, to compare the data of the test group with those of the case-control group. The two tailed Student’s *t* test for either paired or unpaired data was employed as appropriate. A *p* value < 0.05 was taken as statistically significant. 

## 3. Results

The basal, pre-treatment clinical data of the patients are reported on Table 2. There was no statistical difference between the treatment and the control groups in any clinical or biochemical parameter. Although there were some differences between the two groups in drug consumption, no subject modified his/her current therapy (including both hypoglycemic agents and other drugs) during the test period, both in the test and in the control group. The subjects allocated to the ‘functional bread’ group had reported a daily intake of CHO (as bread, breadsticks, cereals or other bread substitutes) of approximately 70 ± 10 g. In the control group, the estimated intake of CHO (starch)-equivalents was similar. From medical records there were no evidence of changes in everyday diet in any of the selected subjects. 

Following the time point defined as ‘start’, clinical and biochemical data were retrieved, after 6.0 ± 0.5 months in the ‘treatment’ group, and after 6.9 ± 0.8 months in the control group (*p* = NS between the two groups). 

In the treatment group, the use of the functional bread resulted in (insignificantly) lower fasting glucose concentrations (by −20.4 ± 16.7 mg/dL, i.e., −1.15 ± 0.92 mmol/L, *p* > 0.05 by paired *t* test), but in significantly lower postprandial glucose concentrations (by −16.4 ± 6.3 mg/dL, i.e., −0.91 ± 0.35 mmol/L, *p* = 0.045 by paired *t* test). The resulting average glucose concentrations were reduced by −17.4 ± 8.0 mg/dL (i.e., by −0.97 ± 0.44 mmol/L, *p* = 0.08 by paired *t* test) (Table 3).

In contrast, in the control group, modest though insignificantly greater fasting (by +2.8 ± 9.9 mg/dL, i.e., +0.15 ± 0.55 mmol/L), post-prandial (by +29.0 ± 10.6 mg/dL, i.e., +1.6 ± 0.6 mmol/L) and mean (by +12.7 ± 6.6 mg/dL, i.e., +0.71 ± 0.34 mmol/L) plasma glucose concentrations were observed. As a result, a significant difference between the two groups was observed (by ANOVA, as interaction effect), as regards both the post-prandial (*p* = 0.011) and the mean (*p* = 0.02) glucose concentrations (Table 3). 

**Figure 1:**
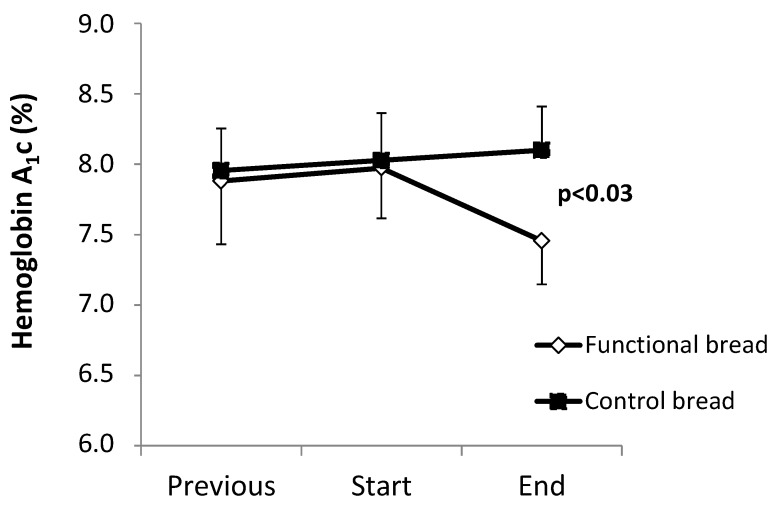
The effect of the functional bread on roughly six-month metabolic control as reflected by HbA_1_c. Hemoglobin A_1_C values (expressed as percent, mean ± SE) in the seven to eight months before (‘previous’), at the beginning (‘start’) and after six to seven months (‘end’) of intake of either the functional bread (open symbols) or the control white bread (filled symbols). The data are expressed as mean ± SE. The *p* value in the figure indicates the significant difference in the changes of HbA_1_c % values, before and following the intake of between either the functional bread or the control bread (by ANOVA).

The HbA_1_c values are shown in Figure 1. Between the ‘previous’ and the ‘start’ observation time points, no difference between the functional and the control bread groups were observed, both groups showing a slight trend (albeit insignificant) towards worsening of HbA_1_c. The observation periods between these two time points were not different between the two groups (treatment group: 6.6 ± 0.6 months; control group: 7.7 ± 1.0 months, *p* = 0.34 between the two).

Following the ‘start’ time point, in the group treated with the functional bread, a −0.52% decrease (as absolute value) of HbA_1_c levels (*p* < 0.028 by the pair *t* test, Figure 1) was observed. In contrast, in the control group HbA_1_c did not change (+0.21% ± 0.13%). As a result, there was a significant difference between the two groups in the post-treatment HbA_1_c values (*p* = 0.027 by ANOVA, interaction effect). Again, there was no difference in the duration of the observation periods between the ‘start’ and the ‘end’ time points, between the treatments and the control group (Table 2). No significant differences between the two groups were however observed in the other clinical and metabolic parameters (Table 4), such a systolic and diastolic blood pressure, total, low-density lipoprotein (LDL) and high-density lipoprotein (HDL) cholesterol. 

Although plasma triglyceride concentrations were greater (*p* < 0.015) in the functional than in the control group at the ‘start’ time point, they did not significantly change in either group between the start and the end periods. Body weight increased by roughly one kilogram in the treatment group (*p* = 0.05 by paired *t* test vs. baseline), but it did not significantly change in the controls (though without significance differences between the groups by ANOVA). 

The data on consumer acceptability are reported in Supplementary material. The overall response was satisfactory, and positive judgments on texture, odor and general acceptance, were delivered, with ranges between 60% and 80%. In contrast, the responses regarding taste were on the whole ‘neutral’, as they did not elicit any prevalence between sweet and salty taste. As a matter of fact, the functional bread was well accepted by the subjects who completed the study. The relatively large drop-out rate (9 out of originally enrolled 20 subjects) was due to reasons other than bread acceptability (see Methods). Most subjects really appreciated the functional bread and are still continuing to use it regularly.

## 4. Discussion

Bread is a staple food, and one of the main sources of carbohydrates and energy worldwide. The diet recommended for a person with diabetes should contain 40%–50% energy as carbohydrates, 15% to 20% as proteins and ~30% as fat. A fiber daily intake of 20–40 g is also suggested [3,12]. Foods with a low glycemic index and/or a low glycemic load would be preferred in diabetes, since they blunt post-prandial hyperglycemia [13,14]. Absolute fiber content [15], type (soluble/insoluble), as well as the presence of active compounds interacting with starch, are important modifiers of carbohydrate digestion and absorption particularly in diabetes. Therefore, the design of a flour containing active ingredients, to be employed for bread preparations, is a ‘hot’ issue today. 

In this study, we report the results of roughly six months’ administration of a home-prepared ‘functional’ bread, made up with a flour enriched with fiber (mainly beta glucan), low in starch and salt, as a substitute for common bread, in subjects with type 2 diabetes in everyday life conditions.

We report that this ‘functional bread’ significantly improved the metabolic control in the T2DM subjects, as shown by significant reductions of glycated hemoglobin, of post-prandial and mean glucose concentrations. In contrast, both HbA_1_c and glucose concentrations did not change in the ‘control’ bread group (Figure 1 and Table 3). Furthermore, in the six to seven months preceding the observation periods, HbA_1_c was nearly stable in both groups (Figure 1). Therefore, these data indicate that the intake of the functional bread was effective in the improvement of metabolic control in type 2 diabetes. 

This study was conducted on everyday life conditions, and based on data retrieval of a limited number of patients regularly visited at the diabetes Centre. Although larger prospective, controlled studies would be required to confirm these findings, our data nevertheless suggest that daily intake of a functional bread could be useful in subjects with T2DM. Notably, in this group the percent reduction of HbA_1_c (−0.52%, as absolute values) was consistent with the concurrent reduction of mean plasma glucose (−17 mg/dL). Indeed, it is well established that a ‘one-point’ reduction of % HbA_1_c is associated with a reduction of 30–40 mg/dL of mean plasma glucose [16]. In addition, the decrease of HbA_1_c values here observed is within the range of changes previously observed after high fiber diets in type 2 diabetes [15].

The efficacy of the functional bread here tested could be due to several, concurring factors. First, as reported in Table 1, the starch content was about half that of the reference, regular bread prepared with refined white wheat flour, on a bread-weight basis. However, no patient reported a *decrease* in bread intake (as grams) with the use of the functional bread. Rather, some of them informed the investigators about a (small) increase in the intake of the functional bread, once they realized that the post-prandial glucose values were improved. Thus, a decrease in the intake (as grams) of the functional bread is very unlikely. Second, the functional bread contained beta glucans, that were absent in the reference bread. Third, the beta glucan/starch ratio in the functional bread was 7.6/100. Such a ratio, although lower than that previously reported to be the optimal value to reduce starch digestion/absorption, could have nevertheless produced a favorable effect [9]. On the other hand, greater ratios than those here tested could further improve post-prandial glucose excursions and the metabolic control in T2DM, should bread palatability be preserved. Fourth, the beta-glucans contained in the functional bread, due to their hydrophilic effect, were probably responsible for its greater water content that, on turn, contributed to bread weight despite the lower starch content. Finally, the total fiber content of the functional bread was nearly double that of the reference bread (Table 1). Therefore, several concurrent variables in the composition of the functional bread could have contributed to its beneficial effects. However, the aim of this study was not to analyze separately the impact of each variable on glucose control in type 2 diabetes. We rather tested the effects of the chosen flour product, available in the supermarket, on glucose control and on sensorial aspects in everyday life conditions. 

A high (>40 g/day) dietary fiber intake is usually recommended to subjects with type 2 diabetes [3,4,5,6,7,8,9,10,11,12]. However, the fiber effect on medium- as well as on long-term metabolic control in T2DM remains controversial [17,18]. A fiber effect had usually been demonstrated when associated to intake of foods with low glycemic index. On the other hand, a high fiber diet may be difficult to be maintained in the long term, because of its poor palatability [19]. In addition, the large gel volume associated to the intake of fibers such as guar gum, despite their efficacy, is often a hurdle in food consumption. In contrast, using beta glucans, the amount of fiber per serving may be increased without either expanding the volume to be ingested or reducing palatability [20]. In this respect, the peculiar composition of the ‘functional flour’ here reported may represent an optimal balance between fiber intake, starch reduction, bread ‘mass’ (i.e., weight), and, last but not least, palatability. 

Beta glucans have been reported to improve satiety and to decrease caloric intake and appetite [21]. These effects are likely driven by an increased viscosity [22], a delay in CHO absorption [23], and a ‘bulking action’ [24]. Beta glucans have also been reported to improve glucose and insulin responses [20]. In addition, the consumption of three grams per day of oat or barley beta-glucans daily was sufficient to decrease blood cholesterol levels [25]. Since beta-glucans reduce the rapidly-digestible starch (RDS) fraction, while increasing that of slowly digestibly starch (SDS) [8], these combined effects would result in a relative increase of resistant starch (RS). However, the beta-glucan-associated reduction of starch digestion rate may also be product- and process-dependent [26]. As a matter of fact, the relationships among beta glucan to starch ratio, starch gelatinization and solubility, and RS formation, are quite complex. At beta glucan to starch ratios below 2 [9], starch solubility was lower at the 1.6/10 than at the 1.1/10 ratio, leading to higher RS and lower SDS values. Our beta-glucan to starch ratio of 0.76/10 probably lead to some increase, although not maximal, of the SDS fraction. Conversely, others reported that a beta glucan to starch ratio of 0.5/10 ratio nevertheless resulted in a significant reduction in starch gelatinization and in its breakdown rate evaluated in vitro [27]. Further research need to be carried out to elucidate these difficult issues.

An association between beta glucan-induced decrease of post-prandial insulin levels, increase satiety and decrease caloric intake, with increase of cholecystokinin, has also been suggested [28]. In our study, neither cholecystokinin nor insulin were measured. Therefore, no conclusions on possible relationships between changes in these two hormones and the observed effects, can be drawn. 

Body weight was not significantly different between the two groups (an analyzed by ANOVA). However, a one kilogram positive change in the treatment group was observed (*p* = 0.05 vs. basal value), whereas weight did not change at all in the control groups. As an explanation of such an unexpected finding, as reported above, some subjects of the treatment group admittedly reported that they had increased the bread intake because they realized that their plasma glucose values were improving, as well as because an increased sense of hunger. Nevertheless, the functional bread was capable to improve the metabolic control, thus independently from any major body weight change. It should also be considered that the raw amount and the energy intake of the diet was generally reported to be unchanged in either group. 

At variance with the improvement of the glycemic control, other clinical and biochemical parameters, such systolic and diastolic blood pressure, total, LDL, and HDL cholesterol, were unchanged by the functional bread intake, being rather stable in both groups. Lack of any effect of blood pressure might be due to the hypotensive treatment of most of our subjects (Table 2). Conversely, despite the reported hypocholesterolemic effects of beta glucan [25,29], in our study lack of any change in cholesterol could be due to the already near-normal values of our subjects, of total as well as LDL cholesterol, (also in respect to target values) (Table 2). Nevertheless, the hypolipidemic effects of beta glucans t in medium-term studies (four weeks) are controversial [29].

Since high-fiber products may decrease palatability [19], our subjects were accurately tested for sensorial responses. The overall response to the functional bread was however satisfactory, with positive judgments on texture, odor and general acceptance recorded in >50% of the subjects, and a virtually neutral response regarding taste (see Table S1 in the Supplementary material). In this regard, other authors [30,31] reported that beta-glucan inclusion improves both the rheological and the sensory properties of bread. 

Although there were some differences in the pharmacological therapy (other than the hypoglycemic agents) between the two groups, with a lower use of statins and ACE-I/ARB, and increased consumption of beta-blockers, in the functional bread group, these differences likely did not affect the results, since no subject modified drug therapy throughout the study. Although statins have been reported to exhibit deleterious effects on plasma glucose [32], plasma glucose was actually unchanged in the control group. Furthermore, the control subjects as well as those of the functional group were on long term therapy with statins. Therefore, statins use per se should not represent a bias in this study.

## 5. Conclusions

In conclusion, regular intake of a low-starch functional bread, enriched with beta glucan fibers, could improve the medium- to long-term glycemic control in type 2 diabetes mellitus in addition to the drugs used to control of blood glucose.

## Figures and Tables

**Table 1:** Flour composition of the ‘functional’ and the ‘control’ (made with refined wheat white flour) breads.

Expressed per 100 g	Functional Bread	Control Bread
Edible part (%)	100	100
Water (g)	43	29
Protein (g)	12	8.6
Lipid (g)	1.2	0.4
Cholesterol (mg)	n.d.	0
Available carbohydrates (g)	31	66.9
Starch (g)	30.2	59.1
Soluble sugars (g)	0.8	1.9
Solubile fiber (g)	2.3	1.46
as beta-glucan	2.3	0
Insolubile fiber (g)	4.7	1.72
Total fiber (g)	7	3.2
Beta-glucan/starch	7.62	0
Energy (kJ)	827	1209
Energy (kcal)	197	289
Salt (mg)	885	1465
Sodium (mg)	354	586

The carbohydrates unit is expressed as g/100 g of the sum of glucose and starch. In this value, fiber is not included.

**Table 2:** Clinical characteristics of the type 2 diabetic subjects, included in either the functional bread or the control group. Data are expressed as means ± SE.

Parameter	Functional Bread Group	Control Bread Group
Number	11	11
Sex (M/F)	4/7	4/7
Age (years)	68.6 ± 1.9	68.0 ± 2.3
Body weight (kg)	72.9 ± 3.6	76.2 ± 4.0
BMI (kg/m^2^)	27.8 ± 1.2	27.9 ± 1.3
Months between measurements	6.0 ± 0.5	6.9 ± 0.8
range	4–9	3–11
*Therapy:*		
Metformin	8	10
Sulphonylureas/Glinides	3	2
Insulin	1	2
Incretins	5	7
Statins	2	8
ACE-I/ARB	2	7
Beta-blockers/Antiadrenergics	4	1
Diuretics	2	4
Aspirin	2	1
Other	7	4

Abbreviations: BMI: Body Mass Index; ACE-I: angiotensin-conversion-enzyme inhibitors; ARB: angiotensin-receptor-blockers.

**Table 3:** Hemoglobin A_1_c (HbA_1_c) (as percent, %), fasting plasma glucose (FPG), post-prandial plasma glucose (PPG) and mean plasma glucose (MPG), in the ‘functional bread’ and in the ‘control’ treatment groups, before (‘start’) and after (‘end’) the observation periods. Data are expressed as means ± SE.

	Functional Bread Group		Control Group		
	Start	End	Start	End	*p* Value
HbA_1_c (%)	7.97 ± 0.36	7.45 ± 0.31 *	8.03 ± 0.34	8.10 ± 0.31	0.0265
FPG (mmol/L)	9.1 ± 0.8	8.0 ± 0.5	8.7 ± 0.7	8.8 ± 0.5	0.29
PPG (mmol/L)	9.2 ± 0.5	8.4 ± 0.6 *	8.7 ± 0.4	9.9 ± 0.6	0.0113
MPG (mmol/L)	9.2 ± 0.6	8.2 ± 0.4 *	8.5 ± 0.6	9.0 ± 0.5	0.02

* Significant difference between the ‘functional bread’ and the ‘control’ groups. The reported *p* values are those resulting from the two-way analysis of variance for repeated measurements, interaction effect.

**Table 4:** Body weight, systolic and diastolic blood pressure, and fasting lipid concentrations, in the groups treated with either the ‘functional’ or the ‘control’ bread, before (‘start’) and after (‘end’) the observation periods. Data are expressed as means ± SE.

	Functional Bread		Control Bread	
Parameter	Start	End	Start	End
Weight (kg)	72.9 ± 3.4	73.9 ± 3.5	77.6 ± 3.3	76.9 ± 3.2
Systolic pressure (mm·Hg)	139.7 ± 5.4	138.6 ± 4.2	137.3 ± 4.9	136.4 ± 6.1
Diastolic pressure (mm·Hg)	75.0 ± 3.4	77.3 ± 3.1	78.6 ± 1.7	79.6 ± 1.9
Total cholesterol (mmol/L)	4.60 ± 0.33	4.55 ± 0.48	4.26 ± 0.16	4.32 ± 0.26
HDL cholesterol (mmol/L)	1.50 ± 0.10	1.46 ± 0.12	1.67 ± 0.08	1.57 ± 0.12
LDL cholesterol (mmol/L)	2.23 ± 0.25	2.36 ± 0.48	2.08 ± 0.15	2.26 ± 0.18
Triglycerides (mmol/L)	1.90 ± 0.29 *	1.60 ± 0.16 *	1.11 ± 0.06	1.08 ± 0.12

* *p* < 0.05 between the ‘functional’ and the ‘control’ bread groups (by ANOVA, group effect). Abbreviations: HDL: high-density lipoprotein; LDL: low-density lipoprotein.

## Supplementary Materials

The following are available online at http://www.mdpi.com/2072-6643/9/3/297/s1.

## Abbreviations

The following abbreviations are used in this manuscript:

CHO	carbohydrates
BMI	Body Mass Index
T2DM	Type 2 Diabetes Mellitus
HbA_1_c	glycated hemoglobin
CV	cardiovascular
GI	Glycemic Index
ACE-I	angiotensin converting enzyme inhibitors
ARB	angiotensin receptors blockers
