# Supplementary material for: A Multifunctional Bread Rich in Beta Glucans and Low in Starch Improves Metabolic Control in Type 2 Diabetes: A Controlled Trial
Source: Nutrients. 2017 Mar 17;9(3):297. doi: 10.3390/nu9030297 (PMC5372960; doi:10.3390/nu9030297)
Supplement: Supplementary file 1 [file nutrients-09-00297-s001.docx]

**Table S1.** Distribution of responses on a hedonic scale of 1–5 (bad to good) for the functional bread group, testing for odor, taste (salty and sweet), texture (soft and crisp), general acceptance*.*

|  | **Assigned Value** |
| --- | --- |
| **FOR ODOUR** |  |
| Dislike very much | 0 |
| Dislike moderately | 0 |
| Neither like nor dislike | 2 |
| Like moderately | 1 |
| Like very much | 8 |
| Total responses | 11 |
| % like responses | 81 |
|  |  |
| **FOR TASTE** | **SALTY SWEET** |
| Dislike very much | 0 0 |
| Dislike moderately | 2 6 |
| Neither like nor dislike | 5 2 |
| Like moderately | 2 1 |
| Like very much | 1 1 |
| Total responses | 11 11 |
| % like responses | 27 19 |
|  |  |
| **FOR TEXTURE** | **SOFT CRISP** |
| Dislike very much | 0 1 |
| Dislike moderately | 2 0 |
| Neither like nor dislike | 1 6 |
| Like moderately | 6 2 |
| Like very much | 1 1 |
| Total responses | 11 11 |
| % like responses | 64 27 |
|  |  |
| **FOR GENERAL ACCEPTANCE** |  |
| Dislike very much | 0 |
| Dislike moderately | 0 |
| Neither like nor dislike | 2 |
| Like moderately | 2 |
| Like very much | 7 |
| Total responses | 11 |
| % like responses | 81 |
